# Supplementary figures and images for: Plant-based red colouration of shell beads 15,000 years ago in Kebara Cave, Mount Carmel (Israel)
Source: PLoS One. 2023 Oct 25;18(10):e0292264. doi: 10.1371/journal.pone.0292264 (PMC10599507; doi:10.1371/journal.pone.0292264)

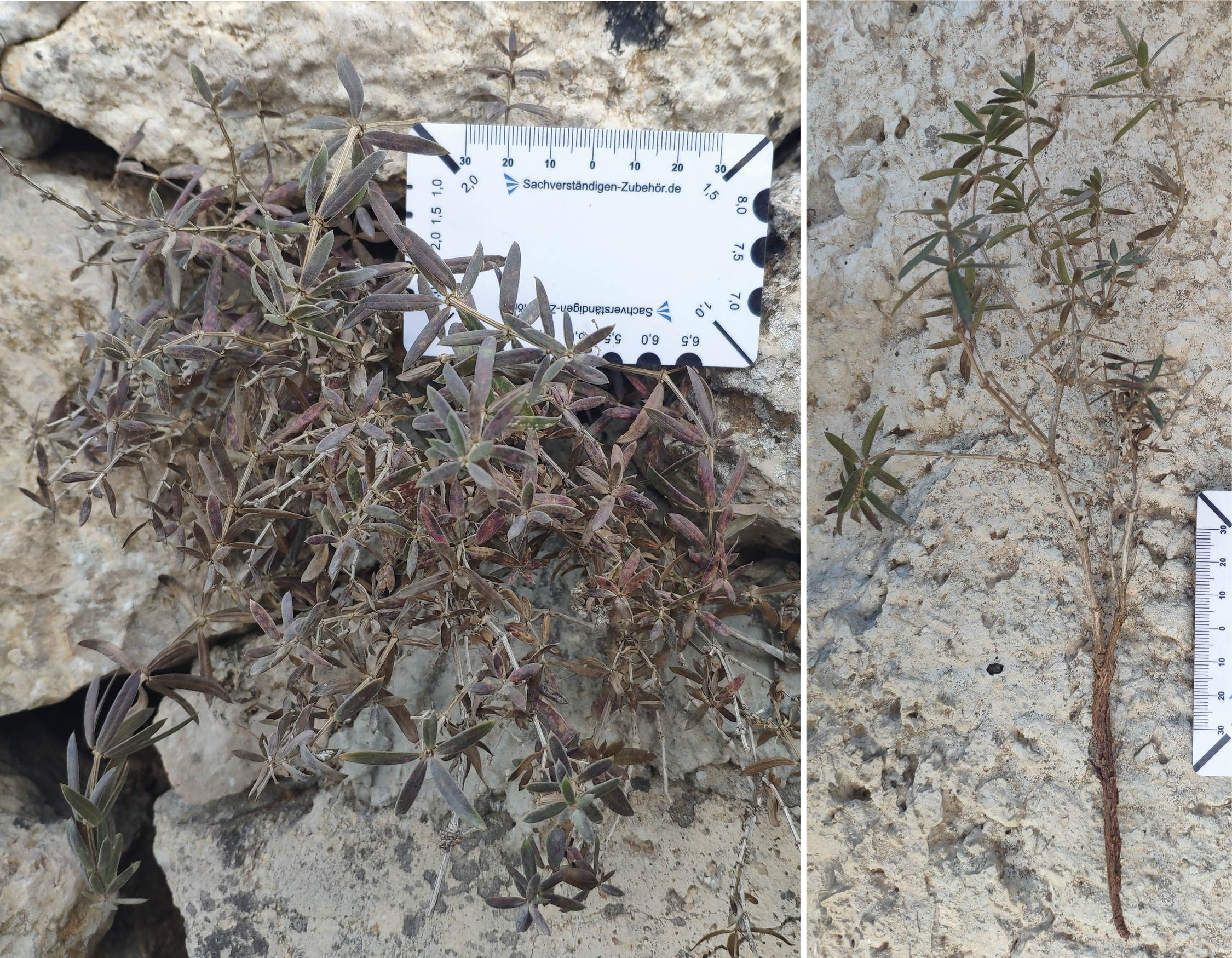

Supplement: S1 Fig — (TIF) [file pone.0292264.s001.tif]

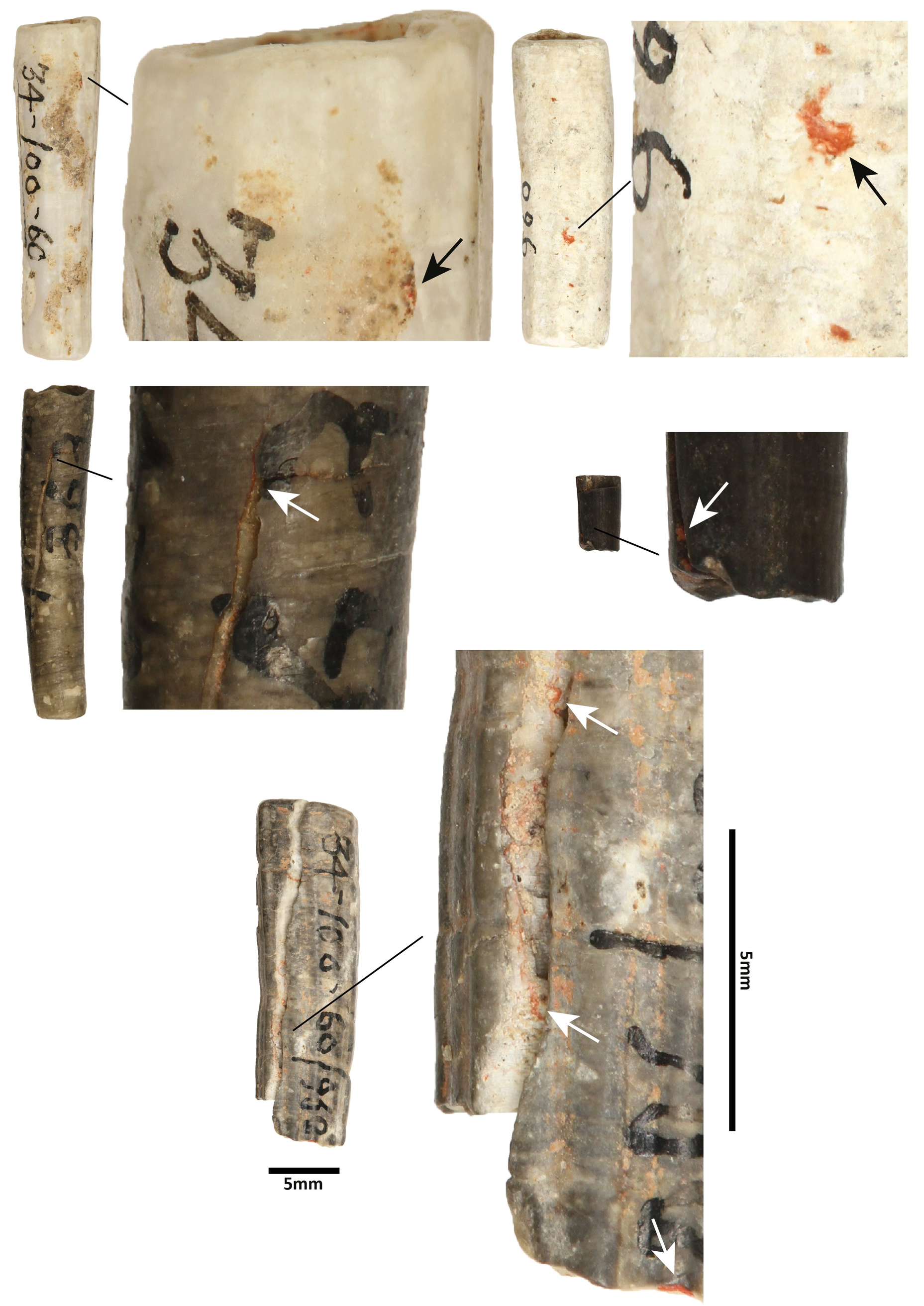

Supplement: S2 Fig — (TIF) [file pone.0292264.s002.tif]
