# Supplementary material for: Plant-based red colouration of shell beads 15,000 years ago in Kebara Cave, Mount Carmel (Israel)
Source: PLoS One. 2023 Oct 25;18(10):e0292264. doi: 10.1371/journal.pone.0292264 (PMC10599507; doi:10.1371/journal.pone.0292264)
Supplement: Attachment — Submitted filename: Response to Reviewers.docx [file pone.0292264.s003.docx]

PONE-D-23-12821

**Plant-based red colouration of shell beads 15,000 years ago in Kebara Cave, Mount Carmel (Israel)**

**Academic editor comments:**

Thank you for submitting your manuscript to PLOS ONE. After careful consideration, we feel that it has merit but does not fully meet PLOS ONE’s publication criteria as it currently stands. Therefore, we invite you to submit a revised version of the manuscript that addresses the points raised during the review process.

This is an interesting study; however, both the reviewers expressed concerns regarding the identification of the materials purportedly used. For this contribution to be considered for publication, you will need to provide a full methodology, and all the relevant data, on an experimental programme covering a wide range of different materials and plants. You will then need to demonstrate, in detail, how you have reached your interpretations on the identification of the materials. The reader will need to be fully confident that they understand how you reached your results, and that, if required, this study is reproducible.

**Reviewers' comments:**

**Reviewer #1:**

This manuscript presents the discovery of an organic plant-based dye on shell beads dating to about 13,000 years cal BP. This discovery is unique and predates the previously known evidence of plant-based dyes from 7,000 years. The data are well presented and the methodology is in adequation with the goals of the paper. However, if the presence of on organic dye is demonstrated, the interpretation that it comes from madder roots would benefit of further discussion. Indeed, it is not clear how this result was obtained and the comparison between the Raman signal of the bead residues and the one of modern madder roots, alizarine and purpurine is not entirely conclusive. The differences between the two types of signals should be discussed in more details.

Response:

The differences between archaeological and reference Raman signals is now detailed in p.12.

The reference signal of other red dyes could also be useful to compare with in order to rule out other hypotheses.

Response:

Other red dyes of plant or animal origin are now detailed and shown in p.7-8, Tab.2 and compared to Kebara dye spectra in Fig.7.

Finally, I recommend a modification of the organisation of the paper. A section “material and methods” could be found before the presentation of the results.

Response:

The description of SEM-EDS and Raman spectroscopy parameters detailed at the end of the manuscript in the “material and methods” section are very technical and we feel that, if they are moved before the results, it could interrupt article flow.

Otherwise, I have just minor comments that are detailed below. The paper is worth publishing with major revision.

Detailed comments

Title: 13 000 years ago instead of 15 000 years ago

Response: p.2 line 72-73 we wrote “layer B provided one of the oldest datings of the Natufian culture with a date of 12,470 ± 180 BP^23^ or 13,315-12,114 **cal BC** (calibrated in OxCal v.4.4^26^).”

12,470 ± 180 BP is the uncalibrated date published by O. Bar-Yosef and A. Sillen (1993). The calibrated date being c. 13,000 before current era (13,315-12,114 **cal BC**) it means c.15,000 years ago (i.e., the beginning of the Natufian culture c. 15,000-11,650 cal BP).

Abstract and page1 line27-38 : red-pigment use started more than 300 000 ka in Africa. It predates modern human emergence in Kenya where it has been found. It is not unique to our species.

Brooks, A.S., Yellen, J.E., Potts, R., Behrensmeyer, A.K., Deino, A.L., Leslie, D.E., Ambrose, S.H., Ferguson, J.R., d’Errico, F., Zipkin, A.M., Whittaker, S., Post, J., Veatch, E.G., Foecke, K., Clark, J.B., 2018. Long-distance stone transport and pigment use in the earliest Middle Stone Age. Science 360, 90–94. https://doi.org/10.1126/science.aao2646

Response: Abstract p.1 line 18-19 we wrote “**habitual use** of red mineral pigments (such as iron-oxide e.g., ochre), by anatomically modern humans started in Africa about 140,000 years ago”

p.1 line 37-38 we wrote “anatomically modern humans probably explains, at least in part, why they started, about 140,000 years ago in Africa**^3^**, to **use habitually** red mineral pigments, such as iron-oxide”

We do not mean that the use of red pigments is unique to our species and that it started only 140,000 years ago. We mean (with reference to Wolf et al., 2018 p.186 “*Habitual use of ochre starts about 140,000 years ago*.”) that the **habitual use** (that Wolf et al., 2018 p.186 consider as “*large assemblages ranging between hundreds and thousands of individual ochre pieces weighing a total of several kilograms*”) of red pigments starts only 140,000 years ago and is associated to our species.

Wolf S., Conard N.J., Floss H., Dapschauskas R., Velliky E. and Kandel A.W. (2018). The Use of Ochre and Painting During the Upper Paleolithic of the Swabian Jura in the Context of the Development of Ochre Use in Africa and Europe. *Open Archaeology*, 4(1), pp.185-205.

<https://doi.org/10.1515/opar-2018-0012>

Abstract and page3 line91: “personal adornments” shouldn’t it be “personal ornaments”?

Response: modified in the whole manuscript

I would recommend to dedicate a special section for the presentation of the materials (line 120-129).

Response: Table S1 in the supplementary materials which present the beads and pendants in detail (Type, sub-type, species, origin, type of identified pigment, position of the residues, etc) is now Table 1 in the main manuscript.

The method section should be placed before the presentation of the results.

In the method section the methods used to identify the molecules is not described. It is a key component of the paper, it must be presented.

Response:

The method used to identify the Natufian colourant are, as stated in the manuscript, Scanning Electron Microscopy with Energy Dispersive X-ray Spectroscopy (SEM-EDS) and Raman spectroscopy whose details of analysis are described in the method section. We added a description and a reference (n°85 and 86) in the method section to explain how those methods works:

85. Goldstein J., (2003). Scanning Electron Microscopy and X-Ray Microanalysis: Third Edition. Springer, 689p.

86. Ferraro J.R., (2003). Introductory Raman Spectroscopy. United Kingdom, Academic Press

Figure 5 – The main peak of the madder roots’ signal is not found on the bead residues’ signal. How do the authors explain such a difference?

Response:

We added several figures and sentences about this point (p.12-13):

“Also, despite being the closest match to the Natufian colourant (Fig.9), the Raman spectra from unprocessed roots of wild common madder (*Rubia peregrina*), slender-leaved madder (*Rubia tenuifolia*) and domesticated common madder (*Rubia tinctorum*) exhibit a strong signal at ca. 1290 cm-^1^ not shown in the Kebara spectra (Fig.9). This strong signal is not shown on the reference Madder Lake (a mixture of purpurin and alizarin made of *R. tinctorum* roots) (Fig.9) and therefore seems to correspond to transformation occurring during the many stages of transformation from fresh root to finished colourant, an observation confirmed by experimental manufacture of alizarin and purpurin lake^46,72^. A direct comparison of the Raman spectra of the Natufian colourant with reference molecules of Purpurin and Alizarin from Sigma Aldrich (Fig.10) show that many of the bands characteristic of this dye are also found in the spectra of these two main anthraquinones molecules originating from the Rubiaceae roots (Tab.2).”

Page 7 line243-249: This should be discussed in more details in the discussion section. Why do the authors think they identified madder dye in the residue on the beads, despite the differences observed between the ancient molecules and the modern madder roots? The reader needs to know more about the distinct hypotheses that could explain these differences.

Response:

We added several sentences and figures (p.9-14):

“The closest match (without being a definite identification), was found with signatures from plant compounds, the anthraquinones (alizarin: C_14_H_8_O_4_ and purpurin: C_14_H_8_O_5_) isolated from the inner root parts of the Rubiaceae family (*Rubia* spp., *Asperula* spp., *Gallium* spp. (Tab.2))^11^.”

“With some representative Raman spectra obtained on the Kebara beads, Figure 8 shows the Raman spectra obtained on the main anthraquinones of Rubiaceae (purified dye of alizarin and purpurin from Sigma-Aldrich and Sennelier) isolated from the roots of domesticated common madder (*Rubia tinctorum* L.), a modern commercial madder lake (mixture of alizarin and purpurin from Kremer Pigmente) and directly measured on the coloured parts of the roots and berries (unprocessed) of wild common madder (*Rubia peregrina* L.) that we gathered near Paris, slender-leaved madder (*Rubia tenuifolia* L.) that we gathered near Jerusalem (Fig.S1) and domesticated common madder (*Rubia tinctorum* L.) bought from an herbalist. The Raman spectra of these products are different because of their nature, *Rubia* spp. roots being unprocessed and commercial references (Sennelier, Sigma-Aldrich and Kremer Pigmente (Fig.8)) resulting from a process of extraction/purification or synthesis that, obviously, would not have been possible in Prehistory. **Similarities can be pointed out between Natufian colourant and modern references, even if there is no perfect match. Although the spectra obtained do not allow the origin of the Natufian colourant to be identified with certainty, everything suggests that it belongs to the family of anthraquinones derived from Rubiaceae plants.** Bearing in mind that natural plant substances are complex mixtures of many molecules, differences could be explained by the characteristics of the Rubiaceae species that were accessible to the Natufians, other anthraquinone molecules such as pseudopurpurin, xanthopurpurin, rubiadin and munjistin could also be present in the Natufian colourant, the relative amounts of which vary with the age of the plant^63^. With regard to the madder plant, even when using a specific plant species, the final composition of a madder colourant can be largely affected by several factors such as cultivation parameters, harvesting and storage conditions of the roots^64,65,66^, effects of colorant extraction process (e.g., drying time, heating temperature, fermentation intensity)^60^ and the method used for colourant preparation^64,67,68^. In addition to the chemistry of the original products, time and the conservation environment may have modified the dye molecules. The taphonomic effects of the acidic conditions of Kebara cave sediment might have transformed the initial colourant (even if it preserved some colour and a molecular signature), knowing also that Raman spectra of dyes are strongly influenced by pH^60,69,70^. Furthermore, up to date, there is no other Raman reference for such an old complex organic dye that would allow us to make a comparison in a similar state of temporal degradation.

For example, the Raman spectra of reference purpurin from Sigma-Aldrich exhibit a strong signal at 1256 cm^-1^ (blue vertical dashed lines at the bottom of Figure 8), not shown on the spectra from Kebara beads, that is characteristic of pseudopurpurin^71^, an unstable anthraquinone that covert to purpurin (decarboxylation: loss of carbon dioxide) over time^71^ or depending on soaking and drying conditions of the roots^60,72^. Also, despite being the closest match to the Natufian colourant (Fig.9), the Raman spectra from unprocessed roots of wild common madder (*Rubia peregrina*), slender-leaved madder (*Rubia tenuifolia*) and domesticated common madder (*Rubia tinctorum*) exhibit a strong signal at ca. 1290 cm^-1^ not shown in the Kebara spectra (Fig.9). This strong signal is not shown on the reference Madder Lake (a mixture of purpurin and alizarin made of *R. tinctorum* roots) (Fig.9) and therefore seems to correspond to transformation occurring during the many stages of transformation from fresh root to finished colourant, an observation confirmed by experimental manufacture of alizarin and purpurin lake^46,72^. A direct comparison of the Raman spectra of the Natufian colourant with reference molecules of Purpurin and Alizarin from Sigma Aldrich (Fig.10) show that many of the bands characteristic of this dye are also found in the spectra of these two main anthraquinones molecules originating from the Rubiaceae roots (Tab.2). The mismatch in relative intensities, which is an essential feature of Raman signatures, does not allow formal identification, but these comparisons highlight the similarities that we have been able to underline.”

Page7 line251: Discussion and conclusion

Response: modified.

Page9 line304: The time gap between the previous earliest known use of madder dyes and the one described in this study is of 7,000 years only.

Response: As detailed in a previous response, the calibrated date being c. 13,000 before current era (13,315-12,114 **cal BC**) it means c. 15,000 years ago. Therefore, the time gap between the previous earliest known use of madder dyes (ca. 6,000 years ago^9,10^) and the one described in this study is of 9,000 years, that’s why we wrote “which are **nearly** 10,000 years older”. We modified the sentence for “**older by 9,000 years** from the first case of Rubiaceae use as a pigment published so far” to be more accurate.

Page9 line329-330: carbon black could come from other organic sources than charcoal. These other sources should be cited too.

Response: modified for “Carbon black (produced, for example, by the incomplete combustion of vegetable matter)”

I would not say that the adding of carbon black increases the saturation of the ochre. I would rather think that it decreases it. For me, this argument is not valid. The adding of carbon black represents a manner of darkening the shade of the ochre.

Response: the sentence “the mixture of organic carbon black (produced, for example, by the combustion of vegetable matter) with the mineral ochre (**which increases the saturation and darkness of the red**)” has been changed for “the mixture of organic carbon black (produced, for example, by the combustion of vegetable matter) with the mineral ochre (**which increases the darkness of the red**)”.

**Reviewer #2:**

I congratulate the authors to raise such an interesting topic.

However, i would recall that the use of natural dyes (and of pigments = of mineral origin) for their antibacterial and antifungal, preservation activities and in some cases even for medicinal purposes.

By adding in the Discussion considerations and an actual brief review of the above-mentioned alternative uses, it might add a more nuanced and pondered interpretation of the data that can drive toward a more complex involvement of the dyes in a far broader range of applications, escaping from the conventional framework of the "only" symbolic use.

Response:

We mention p.2 “Current Prehistoric research recognises red ochre as a universally applied material that serves **a variety of different purposes** from symbolic and ritual display to utilitarian or functional uses, depending on the contexts^3,12^.”

The context here being p.5 “the distribution of coloured residues in different parts of the beads suggests **intentional colouration**”

That is why we emphasize on the colouring purpose of the Natufian plant dye. We can discuss it in comparison with mineral pigment use for colouring purpose that is attested in the Natufian culture. It would be difficult to discuss medicinal purpose given that nothing is known in this field for the Natufian culture.

However, we mention in the discussion p.17 “the aerial parts of the wild madder, stems and leaves, have long been and are still used today in the **traditional medicine** of many cultures^11,59,82,83^ for their **aphrodisiac, antibacterial, anti-oxidant, iron-rich properties**, demonstrated through recent pharmaceutical studies^84^. If indeed we will be able to expand the research, in an endeavour to recover such findings through the study of other artefacts (e.g., groundstone vessels and tools), then **we will enrich our knowledge of the Natufian culture and its practices in new, currently understudied, domains of existence**.”

In summary, we do not rule out a use other than colouring, but further analysis is needed to go further in this field of research.

Out of 16 analysed beads, 6 of them are covered with hematite (pigment). On the basis of preliminary SEM-EDS analysis, the remaining 10 red-stained shell beads were analyzed by Raman spectroscopy, more effective in identifying organic matter, and allowed to identify plant chromophores alizarin and purpurin. The data are interpreted as deriving from the processing of Rubia spp. to get the madder and identified plant source as Rubia tinctorum L..

Response:

Plant source was identified as *Rubia* spp. We modified the manuscript to identify at the family level (Rubiaceae = *Rubia* spp., *Asperula* spp., *Gallium* spp.) given that we need invasive analysis (SERS or HPLC) in order to identify the actual plant species used by the Natufian (alizarin and purpurin anthraquinones are common to *Rubia* spp., *Asperula* spp., *Gallium* spp.). Invasive analysis was not authorized by Israel Antiquities Authority in this study, we hope that the publication of our article will make them reconsider these invasive analysis for a future study.

In order increase the reproducibility of the research, it may be worth to consider other species while building the due reference collection - from which red dyes can be obtained –. For example, Alkanna tinctoria (alkanet or dyer's bugloss), Lawsonia inermis (henna) also present in the region and reported in Egyptian archaeology, Ferula assafoetida, various Galium various species (all available in the Mediterranean rim).

My apologies because the system do not maintain the latin name of the mentioned species.

Response:

A list of potential plant and animal sources has been added in Table 2 and their chromophores Raman and SERS spectra compared to the characteristic bands of Natufian dye in Figure 7.

Along the line of reproducibility, it would be worth that the authors report on their replicative experiment used to obtain the red madder under the conditions and the knowledge putatively convenient/available to the Natufians.

Response:

We did not reproduce red madder under the conditions and the knowledge putatively convenient/available to the Natufians given that, limited by non-invasive analytical methods, we have not been able to identify the actual plant species used by the Natufians. As stated in p.12 “With some representative Raman spectra obtained on the Kebara beads, Figure 8 shows the Raman spectra obtained on the main anthraquinones of Rubiaceae (purified dye of alizarin and purpurin from Sigma-Aldrich and Sennelier) isolated from the roots of domesticated common madder (*Rubia tinctorum* L.), a modern commercial madder lake (mixture of alizarin and purpurin from Kremer Pigmente) and **directly measured on the coloured parts of the roots and berries (unprocessed)** of wild common madder (*Rubia peregrina* L.) that we gathered near Paris, slender-leaved madder (*Rubia tenuifolia* L.) that we gathered near Jerusalem (Fig.S1) and domesticated common madder (*Rubia tinctorum* L.) bought from an herbalist. The Raman spectra of these products are different because of their nature, ***Rubia* spp. roots being unprocessed** and **commercial references** (Sennelier, Sigma-Aldrich and Kremer Pigmente (Fig.8)) **resulting from a process of extraction/purification or synthesis that, obviously, would not have been possible in Prehistory**.”

Also, p.16, we state that “Future studies of experimental manufacture of pigments from various Rubiaceae species coupled with invasive analytical methods on the archaeological beads and tools from Kebara could help identify the actual plant species used by the Natufians and its *chaîne opératoire*.”

To strengthen the interpretation it is also highly recommended to cross-check the replicative data with lab control of the analytical measurements with Standards (i.e. Sigma-Aldrich).

Response:

The spectra that we directly measured on the coloured parts of the roots and berries (unprocessed) of wild common madder (*Rubia peregrina* L.), slender-leaved madder (*Rubia tenuifolia* L.) and domesticated common madder (*Rubia tinctorum* L.) are cross-checked with standards from Sennelier, Sigma-Aldrich and Kremer Pigmente in Figure 8 and 9. The spectra of the Natufian dye is compared with these standards in Figure 10.

These are the main issues that I suggest to strength the impact of an otherwise very interesting research.
