# Supplementary material for: Plant-based red colouration of shell beads 15,000 years ago in Kebara Cave, Mount Carmel (Israel)
Source: PLoS One. 2023 Oct 25;18(10):e0292264. doi: 10.1371/journal.pone.0292264 (PMC10599507; doi:10.1371/journal.pone.0292264)
Supplement: Attachment — Submitted filename: Response to Reviewers.docx [file pone.0292264.s004.docx]

PONE-D-23-12821

**Plant-based red colouration of shell beads 15,000 years ago in Kebara Cave, Mount Carmel (Israel)**

**Journal Requirements:**

Please review your reference list to ensure that it is complete and correct. If you have cited papers that have been retracted, please include the rationale for doing so in the manuscript text, or remove these references and replace them with relevant current references. Any changes to the reference list should be mentioned in the rebuttal letter that accompanies your revised manuscript. If you need to cite a retracted article, indicate the article’s retracted status in the References list and also include a citation and full reference for the retraction notice.

Response:

The reference list is complete and correct. All the references are cited in the text, the figure captions and the method section.

**Academic editor comments:**

The first sentence in the abstract is very broad. Human Universals have defined definitions. I am not sure that this falls into that category. Please reword, to suggest that there is widespread evidence for decoration, or something similar.

Response:

“Decorating the living space, objects, body and clothes with colour seems to be a universal human practice” is now “Decorating the living space, objects, body and clothes with colour **is a widespread human practice**”

I apologise that I did not notice reviewer’s Abstract and page3 line91: “personal adornments” shouldn’t it be “personal ornaments”? Response: modified in the whole manuscript.

You were correct and they are in fact, incorrect – it is adornment, not ornament.

Response:

Modified in the whole manuscript

Language errors have appeared where changes have been made. These all need to be corrected.

EG 41 . correct to plant or animal origin – this is ONE EXAMPLE only. There are a lot more.

47 prehistoric (no capital)

Response:

Modified in the whole manuscript

55 ‘resting on a cushion’ - this needs further explanation. I do not understand what you mean here. What is the evidence for a ‘cushion’?

Response:

According to Fanny Bocquentin's observations during the excavation of Raquefet, the movement of the skeleton in the sepulchral space after the decomposition of the body indicates that in some cases the head of the deceased was placed on a cushion made of perishable material that was coloured red. We have removed this sentence from the manuscript as these observations have not yet been published. F. Bocquentin will be writing a detailed article on the subject in the future.

Your peaks don't align exactly. Raman is fine with one substance/source, but not good for complex organic mixtures See for example Edwards, H.G. and Chalmers, J.M. eds., 2005. Raman spectroscopy in archaeology and art history (Vol. 9). Royal Society of Chemistry.

If this is just madder (*Rubia* spp.) there is arguably a reasonable chance of identifying it using Raman, but even alizarin and purpurin can be difficult to distinguish from each other, unless the pH is fairly alkaline. You have bands which fit with purpurin and alizarin - a mixture of the two - but several of the peaks don't align exactly. There may be reasons for this and the Israeli madder may well be *Rubia tinctorum* but this is based largely on context and inference rather than a high level of correlation.

To be suitable for publication you need to add more important qualifying statements. You cannot go further than suggesting that this might be…. Etc.

Response:

We do not state that we identify the exact plant species that the Natufian used. We state that the Natufian organic colourant is made of a mixture that contain alizarin and purpurin from the roots of one of the Rubiaceae family plants (*Rubia* spp., *Asperula* spp., *Gallium* spp.):

Line 221 “**The closest match (without being a definite identification)**, was found with signatures from plant compounds, the anthraquinones (alizarin: C_14_H_8_O_4_ and purpurin: C_14_H_8_O_5_) isolated from the inner root parts of the **Rubiaceae family** (*Rubia* spp., *Asperula* spp., *Gallium* spp. (Tab.2))”

Line 264-267 “**Similarities can be pointed out** between Natufian colourant and modern references, **even if there is no perfect match**. Although the spectra obtained **do not allow the origin of the Natufian colourant to be identified with certainty**, everything **suggests that it belongs to the family of anthraquinones derived from Rubiaceae plants**.”

Line 296-298 “The mismatch in relative intensities, which is an essential feature of Raman signatures, **does not allow formal identification**, but these comparisons highlight the similarities that we have been able to underline between the Natufian organic colourant and compounds from Rubiaceae plants.” We added this final part to the sentence.

Line 367-369 “**Future studies** of experimental manufacture of pigments from various Rubiaceae species coupled with invasive analytical methods on the archaeological beads and tools from Kebara **could help identify the actual plant species used by the Natufians** and its *chaîne opératoire*.”

For example: Line 157 –Change to ‘suggest’ the presence of…

Response:

Line 157 “Raman spectra (λexc = 458 nm) obtained on Kebara Cave Early Natufian beads and pendants **highlighting** the presence of hematite” is about Fig.3 where archaeological Raman peaks align exactly with reference hematite peaks. In that case, the identification is clear.
