# Supplementary material for: Plant-based red colouration of shell beads 15,000 years ago in Kebara Cave, Mount Carmel (Israel)
Source: PLoS One. 2023 Oct 25;18(10):e0292264. doi: 10.1371/journal.pone.0292264 (PMC10599507; doi:10.1371/journal.pone.0292264)
Supplement: Attachment — Submitted filename: Response to Reviewers.docx [file pone.0292264.s005.docx]

PONE-D-23-12821

**Plant-based red colouration of shell beads 15,000 years ago in Kebara Cave, Mount Carmel (Israel)**

**Academic editor comments:**

There continue to be language issues, please check your ms again and correct these.

Response:

Language issues (correctness and clarity) have been fixed in the manuscript.

I asked that you qualify some of your statements. While I agree that in many cases you are in general not being definitive, you need to change in every case, use of the word 'highlight(ing)' to 'suggest(ing)'. There is a very clear difference; highlighting leaves no room for the possibility that it could be incorrect while 'suggesting' does.

Response:

'highlight(ing)' has been changed to 'suggest(ing)' in the whole manuscript.
